# Comparative Study of Vaccine‐Induced Humoral Immune Response Against Newcastle Disease Virus and Avian Influenza Virus (H9N2) in Different Bird Species of Mashhad Birds Garden

**DOI:** 10.1002/vms3.70191

**Published:** 2025-04-14

**Authors:** Omid BehrouziNasab, Mohammadreza Rouygari, Teimour Tabari

**Affiliations:** ^1^ Department of Clinical Sciences Faculty of Veterinary Medicine, Ferdowsi University of Mashhad Mashhad Iran; ^2^ DVSc. Candidate in Avian Health and Diseases Department of Clinical Sciences Faculty of Veterinary Medicine Shahid Chamran University of Ahwaz Ahwaz Iran; ^3^ Department of Avian Diseases Faculty of Veterinary Medicine University of Tehran Tehran Iran

**Keywords:** AI, avian, immunity, ND, vaccine, wildlife

## Abstract

Viral diseases are the primary cause of disease and economic loss in all bird species. Among viral diseases, Newcastle disease (ND) and avian influenza (AI) have a very special place due to their high morbidity and high mortality. They inflict lots of deaths and economic losses upon poultry and nonpoultry avian species, making them a hot subject in various research. Thus, prevention measures and strategies to control the mentioned diseases are wide. Vaccination is one of the common methods. In this study, we designed a vaccination protocol based on previous and popular methods to evaluate the effect of ND and AI vaccines on the humoral immune response of 14 bird species. We tested 115 birds (sampled twice) with no previous encounter with disease or vaccine. Birds were vaccinated with NDV B1 strain and a bivalent inactivated ND + AI and repeated 5 weeks later. Haemagglutination Inhibition (HI) was used for humoral response evaluation and implemented 5 weeks after each vaccination. Our results showed a double vaccination of ND can boost the immune system significantly, but a single AI vaccination will provide enough protection since the second vaccination did not change the titres significantly, even lowering the titre in one case. There were some discrepancies in results, which further studies should be accomplished to elaborate the details.

## Graphical abstract

The study involved 230 serum samples from various bird species. Birds were vaccinated with ND and AI vaccines, and their antibody titters were measured. ND vaccination significantly increased titres in most species, while AI vaccination had mixed results, with some species showing reduced titters after the second dose. Statistical analyses were performed to determine significance. Further studies are recommended to address discrepancies.

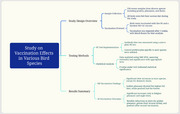

## 1 Introduction

Newcastle disease (ND) is one of the most infectious and highly pathogenic diseases of avian species. It can infect a wide variety of hosts with different levels of immunity against the disease (Ganar et al. 2014). The first appearance of ND dates back to 1926 in Java, Indonesia, which, a year later, was also detected in Newcastle, England (Alborz 2018, Doyle 1927, Kraneveld 1926). The disease is caused by avian orthoavulavirus 1, formerly known as Newcastle disease virus (NDV), belonging to the *Avulavirinae* subfamily of the *Paramyxoviridae* family (Terrestrial Animal Health Code 2014, ICTV 2016, Jeong et al. 2018). To be consistent with the OIE 2014 chapter on infection with NDV, in this paper, we will use the historical abbreviation of the virus–NDV (Terrestrial Animal Health Code 2014). This virus is still a problem worldwide, especially in endemic countries, due to heavy losses that can claim every single one of the whole flock population, reduced egg production, impaired weight gain, decreased fertility and hatchability (Sedeik et al. 2019). NDV has six major structural proteins that are produced following the expression of six RNA genes, 3‐NP–P–M–F–HN–L‐5, which encodes nucleocapsid protein (NP), phosphoprotein (P), matrix (M), fusion (F), hemagglutinin–neuraminidase (HN) and large polymerase protein (L), respectively. HN along with fusion protein is directly involved in virus pathogenicity, providing attachment and fusion of the virus to the host cell and further release of progeny virions from the infected cells (Miller and Koch 2013). Although all strains of NDV belong to one serotype, the fusion gene is used to classify the strain of NDV into class I, 9 genotypes and class II, I–XVIII genotypes.

Infection of NDV induces both humoral and cell‐mediated immunity. Virus neutralization and hemagglutinin‐inhibition antibodies are critical to suppress clinical signs, but this cannot halt the shedding of the virus. Studies have found that T cells are necessary for NDV clearance (Kapczynski, Afonso and Miller 2013). Also, a wide range of cytokines, e.g., IFN‐γ, are involved in defence mechanisms against viral pathogens (Lambrecht et al. 2004).

Immunogenicity of vaccines depends on the production purpose of that specific vaccine. Depending on the situation, production purpose may be based on the amount of produced antibodies, reduced virus shed or decreasing the number of ill or dead birds (Suarez et al. 2020). Also, the route of administration is another factor influencing immunogenicity (Mazija et al. 2010).

Avian influenza (AI) is also another highly contagious and high‐mortality disease. AI virus is a segmented RNA virus that divides into two strain types of low pathogenicity (LPAI) and high pathogenicity (HPAI). Two major antigens contribute to AI pathogenicity: hemagglutinin (H) and neuraminidase (N). Hemagglutinin attaches the virus to sialyloligosaccharide cell receptors and neuraminidase causes cell receptor destruction. As of today, 16 hemagglutinins (H1–H16) and 9 neuraminidases (N1–N9) have been discovered (Swayne, Suarez and Sims 2020). The most common LPAI is considered to be H9N2.

There are restricted zoonoses between birds and humans, and AI is considered one of the main diseases. Although most of the AI transmission focus has been on live bird markets for food, their transmission via pet birds can be a very important route (Boseret et al. 2013). Immunity against influenza engages humoral, mucosal, innate, and cell‐mediated immunity (Koutsakos, Kedzierska and Subbarao 2019). Vaccination is a common act and strategy for controlling AI in poultry, but in pet birds, it is not common; thus, people pay less attention to this matter. But if pet birds can contact wild birds or poultry, the risk of transmission of contagious pathogens, including NDV and AI, rises. Using vaccination to boost the immune system can let us minimize the probability of bird infection, while reducing the risk of zoonosis simultaneously. Thus, in this study, we studied the effect of ND and AI vaccination on several bird species to determine whether it can boost the immune system.

## 2 Material and Methods

### 2.1 Study Design

In this study, we collected 230 serum samples, including native poultry (*Gallus domesticus*) (21), American pheasants (*Phasianus colchicus*) (24), golden pheasants (*Chrysolophus pictus*) (23), Belgian pheasants (a breed of *Chrysolophus pictus*) (16), pigeons (*Columba livia domestica*) (17), guinea fowl (*Numida meleagris*) (12), bronze turkeys (*Meleagris gallopavo*) (12), partridge (*Perdix perdix*) (11), common shelduck (*Tadorna tadorna*) (19), domestic ducks (*Anas platyrhynchos domesticus*) (8), Muscovy ducks (*Cairina moschota*) (12), peafowls (*Pavo cristatus*) (22), buzzard (*Buteo buteo*) (15), and eagles (*Aquila chrysaetos*) (18). All species were fed their normal food during the experiment at the Mashhad Birds Garden.

### 2.2 Vaccination Protocol

All species were vaccinated with eye‐drop live B1 (one drop per bird) followed by subcutaneous injection (0.2 cc per bird) of oil‐adjuvanted bivalent ND + AI vaccine (V4 + H9N2, Razi, Iran) and then repeated the same routine 5 weeks later. Every blood drawn for titre was 5 weeks after each vaccination, and Haemagglutination Inhibition (HI) was implemented at that time. Species had no previous vaccination or any subclinical or clinical disease involvement, so their primary titre was determined as zero.

### 2.3 HI Test

To determine the antibody titre in sera of various species, the beta procedure of the micro‐plate HI test was performed on U‐bottomed 96‐well microtiter plates. In this experiment, we used control erythrocytes of each species for the respective HI. The test was conducted using a continuous ND and AI virus of four HA units.

### 2.4 Statistical Analysis

All data were analysed by IBM SPSS version 27. First, we tested for the normality of data distribution in each dataset by the Shapiro–Wilk test, because each dataset had less than 50 cases. After determining normality, paired *t* test and Mann–Whitney *U* test were implemented for normal and abnormal distribution of data, respectively. *P* < 0.05 was considered a significant value.

## 3 Results

ND vaccination increased the titre of all species significantly (*p* < 0.05), except domestic duck. AI vaccination only increased the titters of Belgian pheasant and eagle significantly. Interestingly, in AI vaccination of some species, the titters were reduced in the second sampling. These species were golden pheasant, guinea fowl, bronze turkey and peafowl. The highest ND on both vaccinations belonged to golden pheasant, while peafowl and Muscovy duck had the lowest ND titre in the first and second vaccinations, respectively. Regarding AI vaccination, common shelduck and partridge had the highest and lowest AI titters in both vaccinations. Also, native poultry joined common shelduck in the second AI vaccination as the highest titre. An overview of ND and AI vaccination results is presented in Tables 1 and 2, respectively.

**TABLE 1:** ND vaccination results (mean titre according to log_2_).

Row	Species	Mean titre 1^a^	Mean titre 2^b^	*P*‐value	Significant?
1	Native poultry	6.81	7.8	0.02	Yes
2	American pheasant	6.41	7.75	0.003	Yes
3	Golden pheasant	7.41	9	<0.001	Yes
4	Belgian pheasant	3.2	6.5	<0.001	Yes
5	Pigeon	6.33	7.8	<0.001	Yes
6	Guinea fowl	5.6	7.3	<0.001	Yes
7	Bronze turkey	6	8	0.037	Yes
8	Partridge	5.3	7.3	0.012	Yes
9	Common shelduck	6.81	8.22	<0.001	Yes
10	Domestic duck	7	7.8	0.058	No
11	Muscovy duck	3	4.3	0.001	Yes
12	Peafowl	2	6.4	<0.001	Yes
13	Buzzard	6.1	8.1	0.003	Yes
14	Eagle	6.37	7.2	<0.001	Yes

^a^ Mean titre 1 is the titre resulted 5 weeks after the first vaccination.

^b^ Mean titre 2 is the titre resulted 5 weeks after the second vaccination.

**TABLE 2:** AI vaccination results (mean titre according to log_2_).

Row	Species	Mean titre 1^a^	Mean titre 2^b^	*P*‐value	Significant?
1	Native poultry	4	4.75	0.347	No
2	American pheasant	4.08	4.16	0.586	No
3	Golden pheasant	3.25	3	0.676	No
4	Belgian pheasant	1.8	2.6	0.001	Yes
5	Pigeon	1.7	2.1	0.197	No
6	Guinea fowl	3.6	3.1	1	No
7	Bronze turkey	2.3	2.2	0.07	No
8	Partridge	1.2	1.8	0.178	No
9	Common shelduck	4.63	4.75	0.170	No
10	Domestic duck	2.6	2.8	0.184	No
11	Muscovy duck	1.8	2	0.07	No
12	Peafowl	2.7	2.45	0.003	Yes
13	Buzzard	1.6	2	0.374	No
14	Eagle	2.5	3	0.033	Yes

^a^ Mean titre 1 is the titre resulted 5 weeks after the first vaccination.

^b^ Mean titre 2 is the titre resulted 5 weeks after the second vaccination.

As you can see, in four species (golden pheasant, guinea fowl, bronze turkey and peafowl) we had reduced titters after the second vaccination, which was significantly reduced in only peafowls.

## 4 Discussion

Our 230 case studies fell into 5 avian families, including *Phasianidae* (native poultry, American pheasant, golden pheasant, Belgian pheasant, bronze turkey, partridge and peafowl), *Anatidae* (common shelduck, domestic duck, and Muscovy duck), *Accipitridae* (buzzard and eagle), *Columbidae* (pigeon), and *Numididae* (Guinea fowl). Producing vaccines for pet birds is very limited due to the complexity of making them for every species as well as the small market size (Heatley, Payne and Tizard 2018). Thus, most of the available vaccines for pet birds are not commercially available and have proven effective or safe by investigation. As a result, almost all vaccines used in nonpoultry birds are prescribed off‐label. In this study, we also used eye‐drop B1 (Razi, Iran) ND vaccine alongside bivalent ND + AI (Razi, Iran) for all species, although it was originally designed for poultry flocks.

Despite all these limitations, our study showed that implementing two ND vaccinations with 5 5‐week interval elevates ND titres significantly. On top of that, the second vaccination also increased the titre of the first vaccination by a significant range in almost every species. Only in one species, domestic duck, second vaccination did not have a significant change in titre (*p* = 0.058), but also in this one, it was very close to being significant (almost 0.008 difference for being significant). As a part of one family, we expect the immune system of its members to be closely related, and the other two members of *Anatidae* (common shelduck and Muscovy duck) had a very significant increase upon the second vaccine intake (*p* < 0.001). Given that our findings are based upon a more limited number of domestic ducks, the results from such analysis should, therefore, be treated with the utmost care. In Stone (1989), the author prepared a vaccine from pigeon paramyxovirus type 1 (PPMV‐1) and NDV strains of LaSota and Ulster. All of the strains kept pigeons safe from morbidity and death, but not from viral infection (Stone 1989). Our study showed that apart from these three strains, V4 strain can also develop a significant immunity. Also, Duchatel et al. (1992) showed that a single dose of vaccination can give pigeons a year‐lasting immunity against ND, irrespective of the maternal antibody.

ND vaccination in pheasants is practised similarly to chickens, and it offers good protection but does not prevent replication. Since the focus of the study was on producing immunity, we did not evaluate virus replication, although the results of pheasants are in good agreement with previous data (Aldous and Alexander 2008). In Siddique et al. (2021), oral administration of the NDV I‐2 strain developed good immunity in ring‐necked pheasants. Also, Jafari et al. (2019) concluded that velogenic ND isolated from Iranian chicken flocks is considered a low pathogenic strain for pheasant and using the B1 NDV vaccine would suffice for reasonable protection, which is barely distinguishable from the results of our study. In one study, vaccinating Guinea fowls with the B1 strain gave the birds good immunity, especially in the presence of maternal antibodies, which confirms the results of our study (Sasu et al. 2020). Vaccination of partridges with B1 strain elevated the number of antibodies significantly, thus offering better immunity (Sousa et al. 1999.).

Ducks possess two important genes in their immune system called retinoid acid‐inducible gene I (RIG‐I) receptor and interferon‐induced transmembrane protein (IFITM), which enables them to fight back ND very strongly (Suarez et al. 2020). As a result, vaccination against ND in ducks is seldom practised, but one study confirmed that duck‐origin velogenic ND strains can cause the disease in ducks and have an important role in its epidemiology (Dai et al. 2014). Thus, maybe we should not take the vaccination of ducks this lightly.

ND vaccination in birds of prey is not commonly practised. In a 2022 study, researchers found an eagle infected with chicken‐origin NDV (Angeliya et al. 2022). Also, previous reports have indicated the presence of ND in raptors (Angeliya et al. 2022, Welch et al. 2019). None of the reports mentioned the raptors having clinical signs, but they can have an important role in the epidemiology of ND. However, vaccination of raptors may be only logical when other species were to be kept among them.

The results of AI vaccination had wide discrepancies. Not only AI titres in the second vaccination did not have any significant change in almost all species, but in some species (golden pheasant, guinea fowl, bronze turkey and peafowl) we observed reduced levels of antibodies, which was significant in only one species (peafowl). Wang, Chen and Yuan (2021) vaccinated peafowls with the H9 AI strain, which resulted in good antibody elevation, which was different from our results. This difference may be due to the different vaccination protocols, since our study had double H9 vaccination. Although Belgian pheasant and golden pheasant are just two breeds of the same animal, *Chrysolophus pictus*, AI vaccination only significantly changed the titre of Belgian pheasant, which may suggest breed difference in AI sensitivity.

Since the second vaccination did not change titres at any significant level, it seems only one AI H9 vaccine will provide enough immunity for protection. To further evaluate the situation, a series of studies should be designed with a longer period of time and an additional challenge with wild virus.

## 5 Conclusion

One ND and AI vaccination for all species seems to elevate the level of antibodies, thus boosting the immune system, but AI does not seem to benefit from the second vaccination. At least we can conclude that AI vaccination in 5‐week intervals is not significantly beneficial to the immune system, and further studies should be designed for a better estimation of vaccine interval in the species.

## Author Contributions

Conceptualization: BehrouziNasab O. Funding acquisition: BehrouziNasab O, Rouygari M and Tabari T. Methodology: BehrouziNasab O. Data curation: Tabari T. Resources: Rouygari M. Investigation: BehrouziNasab O, Rouygari M and Tabari T. Project administration: BehrouziNasab O and Rouygari M. Supervision: BehrouziNasab O. Writing—original draft: Tabari T. Writing—review and editing: BehrouziNasab O, Rouygari M and Tabari T.

## Ethics Statement

The authors have nothing to report.

## Conflicts of Interest

All authors have read and approved the final manuscript. Its contents are solely the responsibility of the authors. All authors declare that they have no conflicts of interest.

### Peer Review

The peer review history for this article is available at https://publons.com/publon/10.1002/vms3.70191.

## Data Availability

There are no data available for this manuscript.
